# Microinterventional system for robot-assisted gonioscopic surgery– technical feasibility and preclinical evaluation in synthetic eye models

**DOI:** 10.1186/s12886-024-03595-3

**Published:** 2024-08-05

**Authors:** Gautam Kamthan, Thijs Meenink, Isabella C. Morgan, Andrew A. Harvey, Jorge L. Lince, Jorrit Smit, Maarten Beelen, James C. Tsai, Marc D. de Smet, Tsontcho Ianchulev

**Affiliations:** 1 New York Eye and Ear Infirmary of Mount Sinai, Research Center, 310 East 14th St, Ste 500 Building, New York, NY 10003 USA; 2 Icahn School of Medicine at Mount Sinai, 1 Gustave L. Levy Place, New York, NY 10029 USA; 3 Preceyes, BV, De Rondom 18, 5612 AP , Eindhoven, Netherlands; 4 Panama Eye Center, Balboa Plaza, Ave. Balboa, Panama, 07127 Panama

**Keywords:** Robot, Precision, Tremor, Glaucoma

## Abstract

**Background:**

Preclinical technical feasibility study of robot-assisted microinvasive glaucoma surgery using a novel ophthalmic robot-assisted surgery system.

**Methods:**

Feasibility was assessed in synthetic eye models in two stages: Stage I, nonimplantable robot-assisted goniotomy; and Stage II, robot-assisted stent implantation using a trabecular bypass stent. Robot-assisted interventions were subsequently compared to the manual approach.

**Results:**

Stage I: Two surgeons completed 10 trials each of ab-interno sectoral goniotomy with and without robotic assistance for at least 3 clock hours using a standard goniotomy knife and more than 10 clock hours of extended goniotomy using a flexible, guided goniotomy instrument. Stage II: Trabecular bypass stent deployment was successfully achieved in 100% of the attempts with and without robotic assistance. Surgical time was recorded and compared between the robotic-assisted and the manual approach.

**Conclusions:**

A system for robot-assisted microinvasive glaucoma surgery can successfully achieve implantable and nonimplantable interventions in the anterior segment. This is the first known demonstration of the feasibility of robot-assisted glaucoma surgery.

## Introduction

Glaucoma is the leading cause of irreversible blindness worldwide. In 2020, there were an estimated 76 million people worldwide with glaucoma aged 40–80 years, with an expected increase to 111.8 million by 2040 [1]. In the US alone, the incidence of open-angle glaucoma increased by 23% from 2000 to 2010. Projections from National Eye Institute data estimate that the glaucoma disease burden will more than double from 2.7 million to approximately 6.3 million by 2050 [2].

Over the last decade, microinvasive glaucoma surgery (MIGS) has overtaken trabeculectomy and tube shunt implantation as the most commonly performed surgical glaucoma procedures [3]. MIGS can be divided into implantable or nonimplantable ab-interno or ab externo interventions. Implantable MIGS involves the use of a permanent device such as a tube or a stent to augment aqueous outflow from the anterior chamber. The main MIGS stent implants (iStent and Hydrus) are designed for trabecular outflow enhancement and are deployed in Schlemm’s canal and the trabecular meshwork (TM) [4]. Nonimplantable MIGS involves excisional removal of the TM using gonio-knives such as the Kahook Dual Blade (KDB) instrument [5, 6].

Unlike conventional glaucoma surgeries, MIGS requires finer movements at a smaller scale, as evidenced in a study by Ang et al. using intraoperative OCT to improve MIGS performance [7]. Gonioscopy is often employed to aide visualization during surgical manipulation and to improve targeting and deployment within microscopic anatomic structures, such as the TM, which is less than 200 microns in size [8, 9]. Malposition and off-target stent deployment remain key challenges associated with gonioscopic surgical intervention [10–12]. Thus, with some of these devices, implantation outside the trabecular meshwork band is possible, and Schlemm’s canal may be missed, impacting the scleral wall and reducing the intended intraocular pressure (IOP)-lowering effect of the implant [10, 11].

**Fig. 1:**
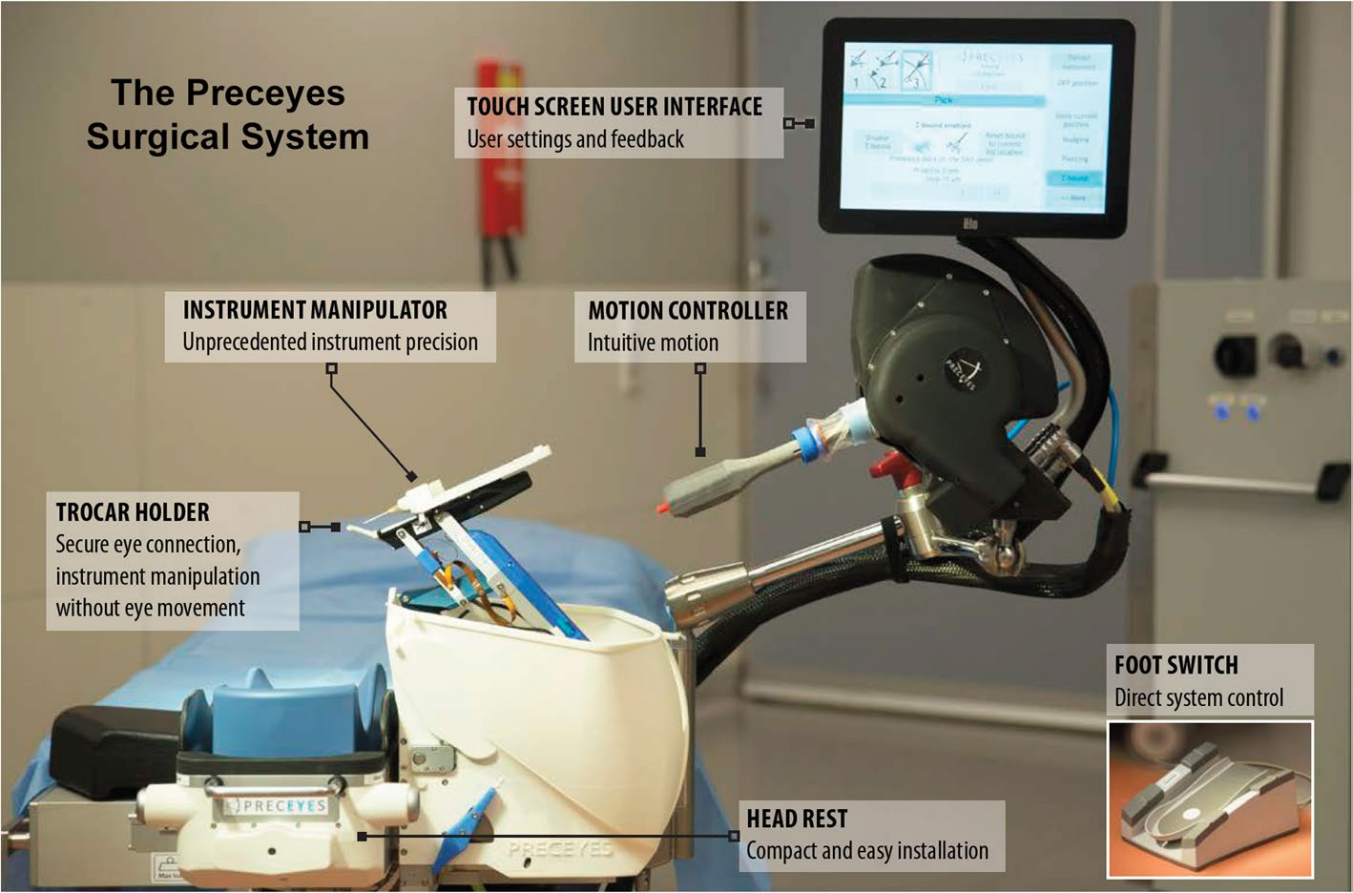
The Preceyes Surgical System. The motion controller has a joystick the surgeon moves to control the instrument manipulator that couples with the patient’s eye. The system can, not only smooth the movements of the surgeon’s hand effectively eliminating the natural resting tremor, but also has built-in safety features to prevent aberrant movements and guidance presets to move the instruments in a predetermined fashion

Conventional surgical knife goniotomy (e.g., KDB) is performed using a rigid trabecular sweep device inserted into the filtration apparatus to strip the TM [6]. Standard gonioscopic knives are rigid and nonguided in their design, which limits their range of motion and trackability within the angle and are usually used to address only approximately 90–120 of the 360-degree trabecular meshwork without repositioning [6]. Robotics with novel instrumentation can address these concerns. With this intent, we developed and reported on the first-in-kind advanced setup for robotic-assisted gonioscopic intervention using the Preceyes Surgical System (PSS) (Preceyes, BV, Eindhoven, NL). This is the first clinical robotic assistive system for intraocular surgery that is CE-marked for ocular surgery, and this system has been used in patients for vitreoretinal procedures (Fig. 1).

### The Preceyes Surgical System

The system allows surgical manipulation with a precision 20-fold greater than that of conventional manual surgery, down to the sub5 µm level [13]. The robotic assistive platform has been backed by decade-long research and validation and has been used clinically in vitreoretinal surgeries, such as the peeling of a macular pucker, subretinal rTPA injections, posterior retinal scanning with an optical coherence tomography (OCT)-based distance sensor, and the use of a range of instruments. An exhaustive discussion of the system’s functions and other robotic platforms still in development is beyond the scope of this study, as this has been detailed in the literature previously [13–19].

In 2020, a new surgical module for the PSS began to develop through collaboration between the New York Eye and the Ear Infirmary of Mount Sinai (NYEEI) and Preceyes B.V. to expand the application of the PSS beyond the retina and to address the need for high precision gonioscopic surgical intervention. The objective of this research was to investigate the *feasibility* of robot-assisted microintervention via two commonly performed MIGS procedures—goniotomy and iStent injection. To this end, several modifications to the existing platform’s software, hardware, and surgeon ergonomics were required and implemented.

## Methods

### PSS modifications for glaucoma surgery

**Fig. 2:**
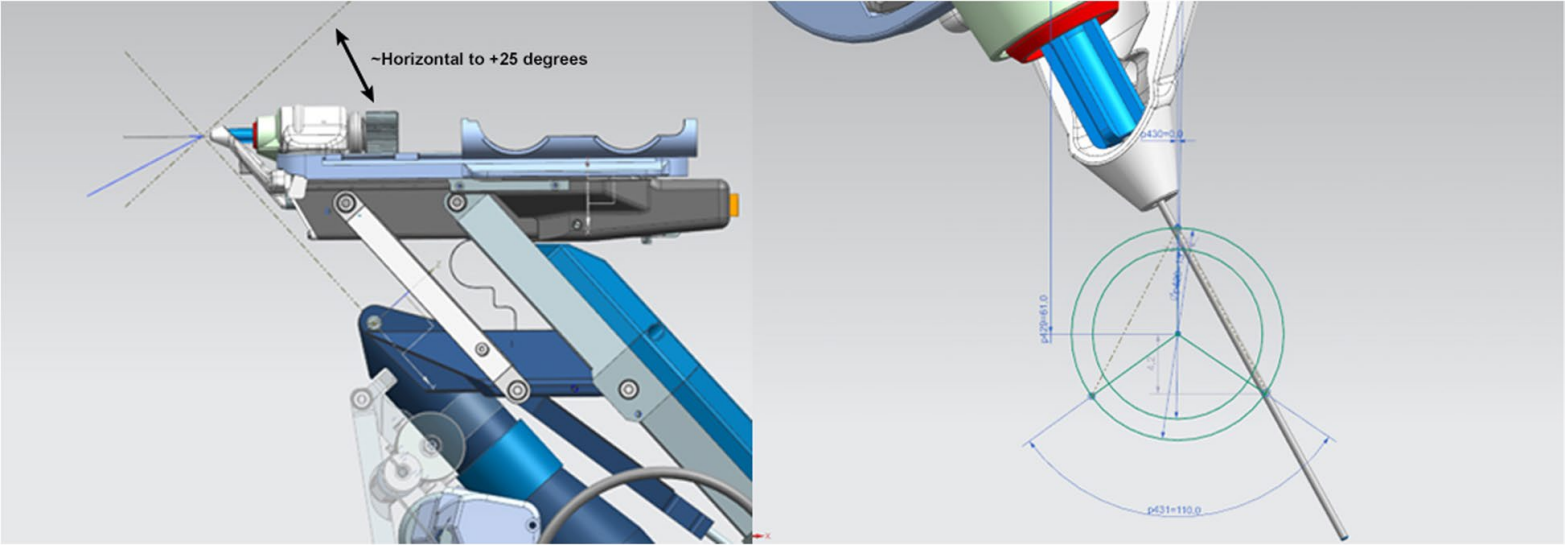
The system was modified to permit a horizontal approach and be able to steepen its angle by 25 degrees (left). A trabecular meshwork sweep of almost 4 clock hours (110 degrees) is possible when in the horizontal position (right, showing system from above). When the eye is tilted, as in many gonioscopic surgeries, the sweep range increases to 5 clock hours (150 degress) (not shown)

Several design elements of the original PSS vitreoretinal platform had to be re-engineered. The PSS was reprogrammed, and precise instrument interfaces were designed to enable a new angular specification related to an anterior segment approach and targeting of anatomical structures within the angle (Fig. 2). The original design did not permit a horizontal approach, as that approach could lead to damage to the crystalline lens during vitreoretinal surgery. For anterior segment surgery, however, the opposite is true, where a horizontal approach is often desired. Therefore, the travel parameters of the PSS arm were adapted to the anatomic design specifications of the anterior segment to prevent iris capture and corneal or lenticular damage, thereby ensuring an atraumatic procedure.

#### Surgeon ergonomics, operating room layout, and angle access during gonioscopy

**Fig. 3:**
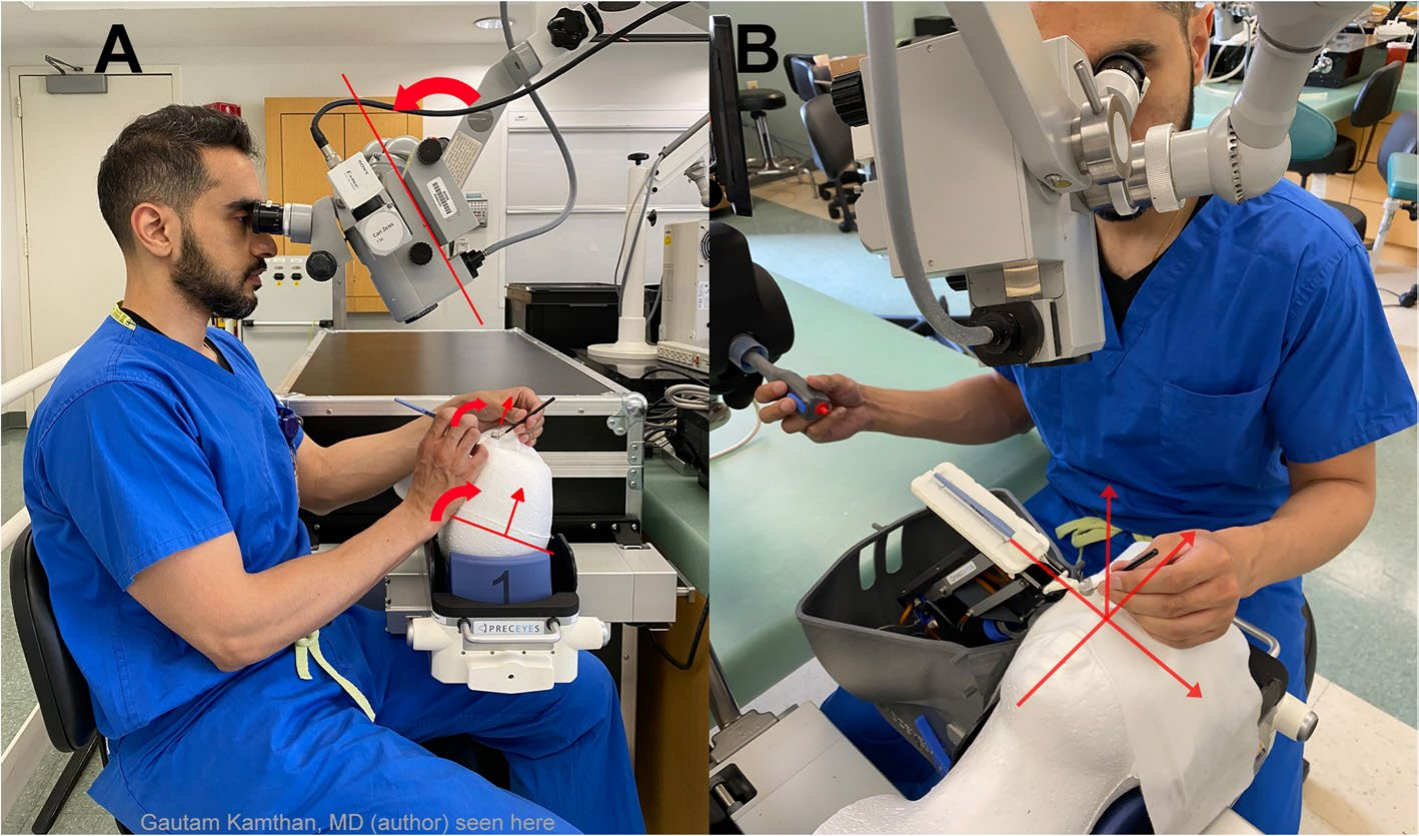
**A **Manual MIGS surgeon and instrument positioning versus (**B**) Robot-assisted MIGS surgeon and instrument positioning. **A** Microscope rotated toward surgeon, patient head and patient eye rotated away from surgeon. **B** Surgeon between PSS and patient’s head with microscope angled toward surgeon, without rotation of patient head or eye. MIGS= Micro-Invasive Glacoma Surgery, PSS= Preceyes Surgical System

**Fig. 4:**
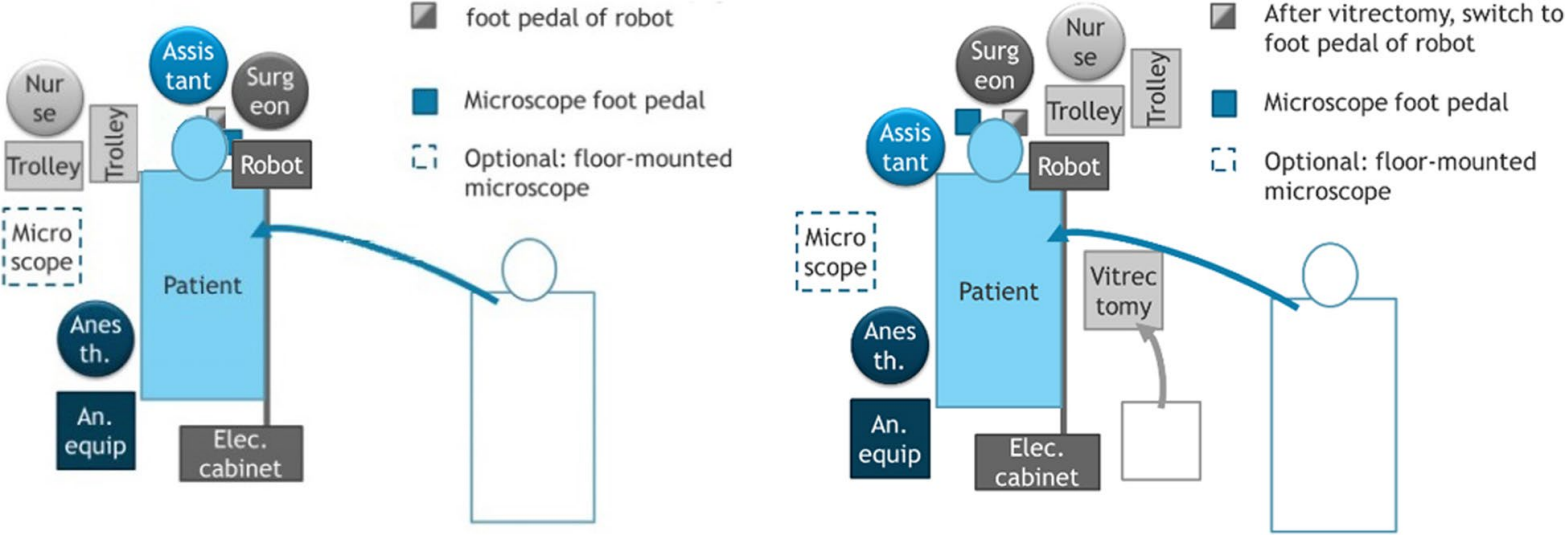
Operating room equipment floor layout demonstrating change from standard vitreoretinal surgery layout to robot-assisted surgery (Left), and floor layout for gonioscopic robot-assisted surgery (Right). Anesth. = Anesthesiologist. An. equip = Anesthesiology equipment

Surgeon ergonomics was modified for comfort, stability, and visualization of angle structures (Fig. 3). In contrast to the original vitreoretinal PSS layout, in which the surgeon sits superiorly to the patient and the PSS is positioned temporally (Fig. 4), the anterior segment surgeon typically sits temporally, especially for MIGS interventions. This presents an ergonomic challenge, as both the PSS and surgeon occupy the same space. In this study, the authors anecdotally found that the most comfortable and stable position was to sit at a 45-degree angle between the patient’s shoulder and head while keeping the PSS in its standard temporal orientation ipsilaterally.

For proper visualization during ab-interno MIGS procedures, there was a concern whether the PSS could simultaneously maintain adequate gonio-access while the surgeon viewed the angle gonioscopically. Traditionally, the microscope is rotated toward the surgeon, and the patient’s head and eye are rotated in the opposite direction (Fig. 3). This results in greater separation between the surgeon and the eye, posing a potential problem for the PSS in bridging such a distance. After readjusting the PSS’s initial design from a vertical approach through a trocar-pars-plana entry for the posterior segment to a horizontal entry through a clear corneal incision for the anterior segment, it was found that rotation of the microscope greater than the standard 30 degrees for MIGS achieved a clear view of the angle, obviated turning the patient’s head and eye, and allowed the PSS to access the angle fully (Fig. 3). Furthermore, there was adequate residual PSS coverage if eye rotation was needed. This was deemed possible as the classic, recommended “en-face” view of the angle for manual MIGS procedures is not needed, since the PSS arm approaches the angle horizontally, regardless of the surgeon’s position and viewing angle.

#### PSS-instrument interfacing and actuation

**Fig. 5:**
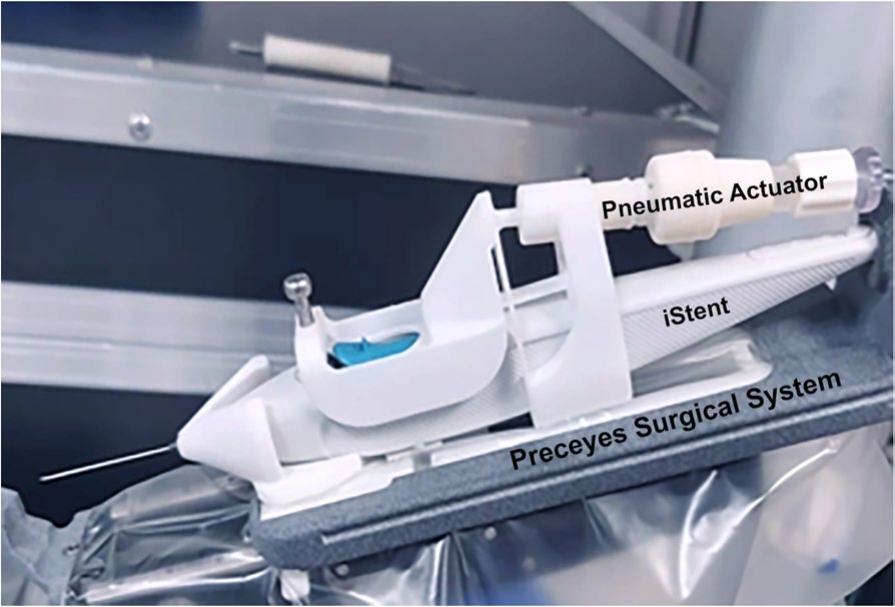
iStent *Inject *W with pneumatic foot-pedal actuator attached onto Preceyes Surgical System

The robotic instrument interface was redesigned to accommodate MIGS devices using an adaptor cradle and port. Instrument adaptors were fashioned for the PSS to hold the MIGS devices while preserving instrument rotation. For the iStent *Inject* W, the sheath can be retracted manually once the device has entered the anterior chamber, but stent implantation requires actuation of a button trigger to achieve precise deployment. Therefore, instrument actuators were designed for the iStent injector to enable robot-assisted foot-pedal firing (Fig. 5). The actuator was fixed onto the iStent device, which could deploy an iStent implant by pressing the foot-pedal that depresses the implantation trigger button on the iStent hand-piece pneumatically. There was no limitation on the number of times the actuator could be triggered.

Pneumatic actuation was selected for the iStent trigger as it was the most technically feasible option, given that the PSS already utilizes pneumatic pressure for forceps actuation. We used a specifically designed for controlling forceps actuator with a stroke/reach of 1,5mm controlled with 1–4 bar. This had the right properties for iStent actuation. To achieve this trigger, a pneumatic instrument body and an adapter to activate the manual instrument was used. The pneumatic actuator is a disposable, robot-specific accessory, and part of the Preceyes accessories. The trigger is activated by gently depressing the foot pedal to increase pressure on the iStent button, until it fires. The foot pedal does not generate pressure, since it is similar in nature to that of a vitrectomy foot-pedal, where the VFI pressure is controlled by the amount pressed. Latency of the pneumatic actuation is not an issue given there is adequate time, stability and control from the PSS. Using an electromechanical actuator such as a step, servo, or piezo motor would have introduced complexity and increased costs. Additionally, it would have been an additional challenge to maintain sterility of the wiring. The pressure towards the actuator is controlled via a pneumatic valve inside the control cabinet. The robot electronic control cabinet is connected to an air pressure source (from a wall socket) and does not generate pressure by itself. The pressure towards the actuator is controlled via a pneumatic valve inside the control cabinet. Lastly, in the existing PSS setup, there were no redundant motors or actuators available to repurpose for iStent actuation.

#### Goniotomy software module

Axes of movement were specifically configured for motion in the anterior segment, and a “Goniotomy” software module was created, where anterior/posterior movements and those along the longitudinal axis of the instrument were limited or neutralized. Rotational movements were also significantly slowed to minimize locking of the tip of the goniotomy knife within Schlemm’s canal or damage the surrounding tissues during the goniotomy sweep. Therefore, using the goniotomy module facilitated a smooth uniform curvilinear sweep in Schlemm’s Canal.

### Selection of MIGS devices and model anterior chamber

**Fig. 6:**
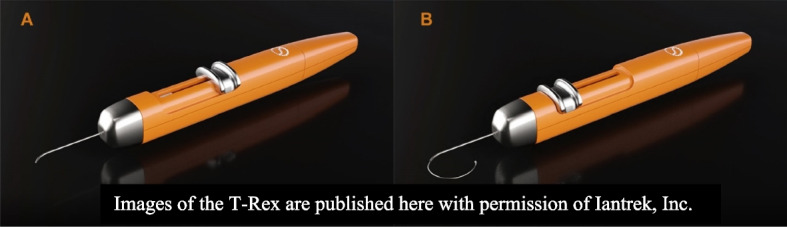
Micro-interventional flexible trabeculorrhexis device (T-Rex, lantrek, Inc.) (**A**) Retracted (**B**) Extended

Two goniotomy technologies for nonimplantation-based MIGS interventions were used. The first goniotomy device used was a standard goniotomy knife (Kahook Dual Blade Glide, New World Medical, Inc., Rancho Cucamonga, CA). The second is a flexible nitinol filament for continuous microinterventional trabeculotomy (T-Rex, Iantrek, Inc., White Plains, NY). This approach was used to complete a 180-degree curvilinear, single-pass, flexible microinterventional goniotomy (FMG) with robotic assistance (Fig. 6).

We chose the most commonly used ab-interno trabecular stent (iStent *Inject* W, Glaukos, Inc., San Clemente, CA) as our implantable MIGS device. We coupled the injector to the Preceyes platform to enable intraocular actuation and trabecular deployment.

SimulEYE KDB/TrabEx and SimulEYE iStent model eyes were used (SimulEYE, Westlake Village, CA), as these are standard training models for MIGS with the necessary anatomical structures, including peripheral clear corneal incisions, an iris, a trabecular meshwork, and an angle with a Schlemm’s canal that can be cannulated or stented for 360 degrees. In these model eyes, the corneas provide a life-like experience and require delicate manipulation to avoid any deformation while using a gonioprism. Animal or human cadaveric eyes were not used because they can violate hospital safety and infection control protocols for the PSS since it will be used in the future for in vivo surgery. The model eyes were placed in a 3D printed model globe for fixation into a Styrofoam dummy head. This allowed for independent rotation of the head and eye, as would be expected in vivo. Surgical drapes were not applied at this stage as this was a feasibility study, and it was not anticipated that they would affect surgical results using the PSS, given the experience of draping the system for vitreoretinal surgery.

### Experimental design

We divided the study into two stages: nonimplantable MIGS goniotomy using the KDB Glide and T-Rex into Stage I and MIGS trabecular stenting with the iStent *Inject* W device into Stage II. To evaluate the robotic-assisted platform and compare its performance to that of manual surgery, two anterior segment surgeons experienced with MIGS were asked to perform ten attempts manually and ten with the PSS for the KDB, T-Rex, and iStent each. Thus, 60 total surgical manipulations were performed per surgeon (30 manually and 30 with the PSS) for a total of 120 surgical procedures across both surgeons in this study. Warm-up attempts and randomization were not included in this feasibility study because the qualitative performance of the procedures was the focus, not a quantitative assessment of the finesse or a demonstration of improved performance of the robot compared to manual performance.

All angle-based procedures were attempted while holding a Swan–Jacobs gonioscopy lens in one hand for visualization. This lens was chosen because it is the most commonly used lens at our institution and provides adequate angle visualization while permitting sufficient access through the corneal incision. The “damage” to adjacent structures was recorded to evaluate the prototype’s safety as an additional outcome measure. As this study used synthetic eye models, “damage” is defined as any visible, permanent distortion of the original structure of the model. This “damage” was visualized using gonioscopy and a microscope. The iStent diameter is 230 µm, so anything within 1 iStent diameter was disregarded. Anything above roughly 1 diameter was considered “damage”.

In our study, *laboratory feasibility* was defined as completion of the procedure at least once by one surgeon. The main outcome measures were the feasibility of sectoral goniotomy, extended goniotomy, and stent implantation using the PSS we modified for MIGS as described above. We aimed to assess feasibility not only once but also through 20 attempts across two surgeons for each procedure to evaluate *real-word feasibility*, a more practical measure of the success of our prototype’s modifications. We also compared the feasibility of robot-assisted attempts to standard manual attempts as a benchmark for understanding the ease of use of robotic assistance for these procedures. These comparisons were not intended to indicate the clinical efficacy of the robot over manual surgery. This topic is beyond the scope of this study. Surgeons visually evaluate successful entry into the anterior chamber (AC) through the cornea without impacting the iris, cornea or lens structure and the ability to visualize and engage the trabecular structures without aborting the procedure.

#### Stage I goniotomy

Stage I consisted of the main outcome measure of at least three clock hours of successful goniotomy (sectoral goniotomy) prior to disengagement or aborting the procedure using the KDB and at least ten clock hours (extended goniotomy) using the T-Rex. We recorded the total number of clock hours of goniotomy achieved for each attempted goniotomy. Each surgeon carried out ten manual and ten robotic assisted procedures per device. Surgical time was recorded from the point where the instrument was positioned inside the anterior chamber over the middle of the pupil, the goniotomy achieved, and the instrument returned to the original starting point over the pupil.

#### Stage II iStent implantation

The surgeons were assessed for the main outcome measure of their ability to perform an atraumatic and unimpeded implantation of the iStent. Ten implantations were executed manually and with robotic assistance. Surgical time was recorded from the point where the instrument was positioned inside the anterior chamber over the middle of the pupil, the iStent implantation achieved, and the instrument returned to the original starting point over the pupil.

## Results

### Stage I: nonimplantable MIGS

**Fig. 7:**
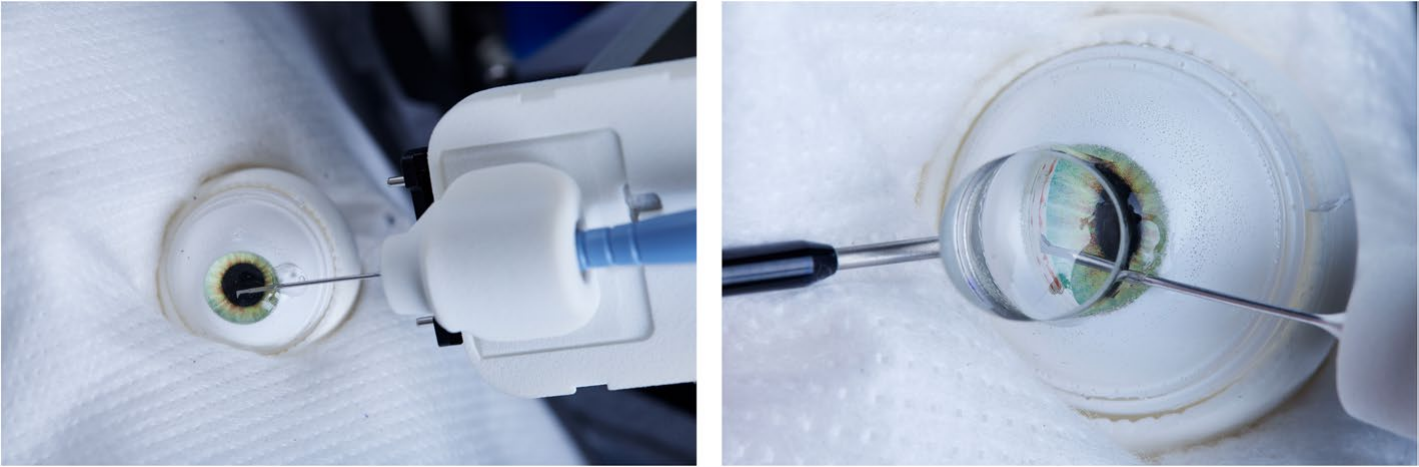
Preceyes Surgical System (PSS) holding a Kahook Dual Blade and successfully entering the model eye, without damage to adjacent structures (Left). PSS crossing the pupil and positioned in angle for a gonio-sweep (Right)

A goniotomy was performed in each eye successfully using the KDB in all robot-assisted interventions. In 100% of cases (20 out of 20 attempts), 3 clock hours of goniotomy (sectoral goniotomy) were performed using the robotic-assisted module. This outcome is similar to the success achieved with manual intervention, where sectoral goniotomy was accomplished in 20 out of the 20 cases (ten per surgeon) (Fig. 7).

In addition, FMG was achieved with robotic assistance in all 20 cases, and in all cases, at least 10 clock hours of extended goniotomy were surgically accomplished. No manual FMG was done because the FMG interface was specifically designed for robotic actuation; this in turn is a limitation in this study. We wanted mainly to evidence that FMG is possible using the PSS. No cases of extended goniotomy were possible manually or with robotic assistance using the KDB (Table 1).

**Table 1:** Stage I—Robot-assisted vs manual goniotomy results

	Sectoral Goniotomy (3 clock hours)		Extended Goniotomy (> 10 clock hours)		Damage to Intraocular Structures	
Intervention	Successful Attempts	% of Attempts	Successful Attempts	% of Attempts	Attempts	% of Attempts
PSS^a^ using conventional gonio-knife	20/20	100%	N/A	N/A	0/20	0%
Manual using conventional gonio-knife	20/20	100%	N/A	N/A	0/20	0%
PSS using FMG^b^	20/20	100%	20/20	100%	0/20	0%

Clock hours performed with a conventional gonio-knife with and without the PSS, PSS and a flexible microinterventional goniotomy instrument, and the damage to surrounding structures

^a^*PSS* Preceyes Surgical System

^b^*FMG* Flexible Micro-Interventional Goniotomy

No cases of damage to surrounding intraocular structures occurred during any of the procedures across either surgeon.

### Stage II: implantable MIGS

Implantation of stents was successful in all eyes attempted and with each device, manually and with robotic assistance (Table 2). The TM of the model eyes was examined following the simulated surgery to evaluate the implantation sites. In both manual and robotic-assisted procedures, the stents were successfully implanted into the TM. Twenty out of twenty total implantations across both surgeons achieved successful deployment with the robot-assisted approach. This outcome was similar to the success achieved with manual implantation, where all stents were also implanted on-target. However, in 40% of the manual implantations, the peri-implantation sites exhibited noticeably different amounts of “trauma” extending to an area greater than the diameter of the head of the iStent W. We measured this area in terms of “stent diameters” to quantify the amount of “tissue” disruption (as this study was conducted in synthetic eye models, no actual trauma occurred to any biological tissue— “trauma” and “tissue” can be interpreted as referring to structural damage to the eye model as a result of the procedure). Other manual cases had implantation site trauma limited to an area within 1 stent diameter. This was not associated with any stent dislodgement or instability, nor was there a difference between the two surgeons. No robotic iStent implantations showed a greater than 1 stent diameter area of tissue disruption.

**Table 2:** Stage II—implantable micro-invasive glaucoma surgery with and without robot-assistance

	On-Target iStent TM^b^ penetration/entry (within TM band)		Off-Target penetration/entry (completely outside TM)		Peri-implantation site trauma or tissue disruption > 1 stent diameter	
Intervention	Successful Attempts	% of Attempts	Attempts	Percent	Attempts	% of Attempts
^a^PSS iStent Implantation	20/20	100%	0/20	0%	0/20	0%
Manual iStent Implantation	20/20	100%	0/20	0%	8/20	40%

Robot-Assisted vs Manual iStent *inject* W results, on-target vs off-target implantation, and the amount of peri-implantation site trauma

^a^*PSS* Preceyes Surgical System

^b^*TM* Trabecular Meshwork

## Surgical time

Surgical time was also recorded for each trial (Table 3). This study was not designed to assess surgical efficiency as this was a feasibility study. Nonetheless, some observations from the data were notable. Firstly, all procedures were completed in less than 60 s, whether performed manually or robot-assisted. The average robotic trial for all tests lasted approximately 15 s whereas the average manual trial was approximately 9 s. Secondly, after 5 trials with the PSS for each instrument, the procedure time decreased for the subsequent 5 trials. For example, the average surgical time for the first 5 cases with each instrument using the PSS was 17.6 s, while the average for the last 5 cases was 12.50 s. Lastly, some robotic trials involved error from the assistant when changing the settings to activate, modify, and deactivate the prototype software module. This translated to a longer surgical time and affected the average time for the PSS-assisted cases.

**Table 3:** Surgical time – implantable micro-invasive glaucoma surgery with and without robot-assistance

Surgical Approach (amount)	Maximum Time (s^b^)	Minimum Time (s)	Average Time (s)	First 5 cases Average (s)	Last 5 cases Average (s)	p value of first 5 and last 5 trials
^a^PSS-assisted (60)	59.49	5.92	15.09	17.61	12.50	0.0009
Manual Approach (60)	15.6	4.28	8.73	9.09	8.37	0.13

Robot-Assisted vs Manual surgical time

^a^*PSS* Preceyes Surgical System

^b^*s* Seconds

## Discussion

Our results successfully demonstrated the feasibility of robot-assisted gonioscopic intervention in a high-fidelity, preclinical synthetic model for MIGS. In our feasibility study, laboratory feasibility was defined as the case in which one surgeon performed the procedure at least once. If a procedure is demonstrated once, then that proves that the procedure is feasible. We chose to assess this over multiple attempts by multiple surgeons, however, to demonstrate real-world feasibility. Repeating the procedure with multiple surgeons better establishes that the feasibility of performing the procedure is not due to chance. This outcome was objectively achieved in each stage. In Stage I, sectoral and extended goniotomy were achieved in all attempts. In Stage II, the modified PSS demonstrated successful stent implantation. These nonimplantable and implantable gonioscopic interventions were successful at equivalent to manual surgical techniques. When coupled with advanced microinterventional instrumentation, the PSS was able to perform extended, continuous > 10 clock hours of goniotomy, illustrating the synergistic potential of high-precision robotic assistance and next-generation instrumentation. This opens up insight into the range of potential benefits associated with the PSS interface that could be applied to glaucoma surgery for next-level intraocular surgical navigation with micron-level precision, tremor stabilization and automation.

With our study, we empirically validated key aspects of robot-assisted surgical manipulation in the anterior segment. We were able to couple, advance, and successfully control conventional surgical instrumentation in the anterior chamber with similar atraumatic facility and anatomic outcomes as seen in the manual paradigm. We leveraged and tailored many of the robot-assisted elements of the retina system to the new positional environment of the anterior chamber and the gonioanatomy. In addition, we were able to demonstrate that both implantable and nonimplantable MIGS are amenable to robot-assisted interfaces, including surgeon ergonomics, anatomic access and tissue manipulation. While this study was not designed to quantitatively compare the accuracy of robotic-assisted versus nonrobotic interventions, micron-level motion precision and tremor stabilization were inherently built into the robot-assisted interface and were part of the interventional technical evaluation. These parameters of the robot-assistive system were previously evaluated in the retina experimental validation of the PSS [14].

The results also demonstrated the feasibility of robot-assisted actuation in glaucoma surgery. Stent implantation requires device actuation for the deployment of hardware, using the foot pedal in this setup. The feasibility of robot-assisted actuation is an important aspect of the robot-assisted paradigm as more actuation-driven instruments enter surgical use.

The PSS has built-in tremor stabilization, and its benefits became apparent during simulated gonioscopic intervention. With the PSS, it was surgically possible to pause and “park” the instrument at any stationary point. This approach allowed both the use of a conventional knife and flexible microinterventional goniotomy to be controlled. PSS tremor stabilization was particularly helpful with ab-interno MIGS, which requires a two-handed technique, one hand conducting intraocular manipulation and the other maintaining the angular tilt of the globe and the goniolens. With respect to the dominant surgical hand, the PSS was able to eliminate unintended movement and tremors, which reduces the source of manual variability to only the nondominant hand. This was particularly notable during iStent implantation, when targeting the microscopic band of the trabecular meshwork was mostly tremor free. Whether tremor elimination is a determinant of better surgical outcomes remains to be validated in clinical practice; in fact, in our study, stent deployment success was equal in both manual and robotic-assisted interventions.

The PSS’ software also provided other mechanisms to enhance precision that were not initially obvious. For example, when performing a goniotomy, the “goniotomy” mode enables the PSS to perform the sweep in the angle but prevents it from penetrating deeper because of the limitation of the mode to movement only in specific axes. In the future, similar semiautomation methods can be used to develop single-pass continuous trabeculotomy or trabeculorrhexis procedures and circumscribe a precise paralimbal arc for trabecular tissue excision.

For iStent implantation, deep implantation can render the stent ineffective and cause unnecessary bleeding, thereby obscuring the view for confirmation of correct implantation and sequential stent implantation. To avoid this, we used the PSS’s built-in “nudging” feature to our advantage. This feature permits the surgeon to advance the instrument at user-specified micron-level increments. In this mode, the tip only advances longitudinally along the device’s axis, while any side-to-side, elevation/depression, or rotational movements are zeroed. The benefits of this approach include the following: first, this approach precludes the risk of deep or superficial implantation; second, the tip enters and exits the TM exactly along the same axis, thereby preventing inadvertent damage to surrounding tissues—typically caused by the surgeon’s tremor.

Currently, connecting the instrumentation to the PSS requires specific coupling for each instrument. Modification of the iStent adaptor involved significant iteration because the injector is much larger than the KDB and T-Rex handpieces. Consequently, a new holder had to be designed to accommodate the iStent needle—a custom adaptation that may be eliminated in the future with a more universal adaptor that fits various devices.

While the results of this early study were promising, further work is needed to optimize the setup. For example, the TM of these model eyes is obviously demarcated. In human eyes, there is great variability in TM pigmentation, which could affect the success rate of trabecular MIGS. There are other commercially available model eyes, such as the Bioniko models, that have lighter pigmented TMs, which may affect the results. Additionally, the corneas of the model eyes are more rigid than in vivo corneas. During surgery, corneal deformation is a major hindrance to the surgeon, causing an obscured view and leakage of the viscoelastic from the chamber. As a result, it was not possible to evaluate the effects of possible forces acting on the corneal wound during the procedure. Furthermore, the setup does not involve the patient’s head or eye movement as a confounder, which is an important factor during surgery. Last, this study involved only two surgeons. Future work will need to test more data to fully evaluate the generalizability of the findings.

The PSS software was configured to develop the Goniotomy module and enable a simplified yet precise robotic-assisted goniotomy. This feature will need further optimization as it currently assumes a spherical surface, meaning that the movement of the instrument tip proceeds along a circular arc. However, Schlemm’s Canal is typically elliptical [9]. For this study, this geometric discrepancy did not produce any identifiable adverse effects. Future iterations could benefit from modifications providing computer vision or robotic haptic/tactile feedback versus possible big data artificial intelligence (AI) analysis of Schlemm’s Canal morphology to aid in guiding the instrument tip. Such advancements would be advantageous in many areas, including iStent implantation. For example, simply adding intraoperative OCT guidance can aid surgeons in identifying necessary structures, especially when they are difficult to visualize via intraoperative gonioscopy. Finally, our study evaluated the feasibility of the setup with only two surgeons. Future studies need to assess more surgeons to understand the generalizability of the findings and the system modifications.

An unexpected finding in this study was the apparent difference in peri-implantation site “trauma” of the TM between robotic-assisted and manual iStent implantation. We assessed this difference using “stent diameters” to quantify the amount of “tissue” disruption. This was deemed critical because the associated trauma during implantation may be a cause of stent malposition. For example, if the implantation site tissue is distorted, the stent may dislocate, rendering the stent ineffective. Qualitatively, we observed that robotic assistance enabled us to more accurately implant the stents where it was intended, i.e., the center of the TM. While the manual approach succeeded in implanting stents within the TM, not all the stents were centered consistently. We are further analyzing this to quantitatively assess the difference, which will require further study.

Operating time is also a consideration when introducing new hardware for surgical interventions. We demonstrated that after 5 trials with the robot, the surgeon’s procedure time significantly improved. This was attributed to a learning curve with the robot. As the surgeons are both experienced MIGS surgeons, a learning curve for the manual approach was not seen, since this approach was most like the current in vivo technique. There was also the need for an assistant in this prototype module that affected the surgical times by introducing an opportunity for human error and delay when the assistant changes settings on the touchscreen. This could be improved by, for example, modifying the software to enable foot pedal or hand control for the surgeon to switch between the anterior segment goniotomy module and the standard anterior segment module. Such a change could reduce the operating time and even obviate the need for an assistant. Thus, further optimization of the prototype software module and robotic integration into the anterior segment surgeon’s paradigm will be necessary and may improve surgical efficiency. Future studies of surgical times, such as the recent evaluation of robot-assisted epiretinal membrane peeling with the PSS [20], will be valuable for this novel anterior segment module.

This study highlights not only the feasibility but also the potential of robotic-assisted intervention for intraocular glaucoma surgery. Future studies need to be performed to evaluate any benefits of such robot-assisted interventions over manual performance. This study is a first step toward a larger paradigm shift toward high-precision, microinterventional ophthalmic robotics, the value and clinical utility of which are just beginning to be understood.

## Data Availability

All data generated or analysed during this study are included in this published article.

## Abbreviations

IOP: Intraocular pressure
MIGS: Microinvasive Glaucoma Surgery
PSS: Preceyes Surgical System
NYEEI: New York Eye and Ear Infirmary
KDB: Kahook Dual Blade
MAUDE: Manufacturer and User Facility Device Experience
ERM: Epiretinal membrane
AI: Artificial intelligence
OCT: Optical coherence tomography
FMG: Flexible microinterventional goniotomy
